# A Micro-Thermal Sensor for Focal Therapy Applications

**DOI:** 10.1038/srep21395

**Published:** 2016-02-26

**Authors:** Harishankar Natesan, Wyatt Hodges, Jeunghwan Choi, Sean Lubner, Chris Dames, John Bischof

**Affiliations:** 1 Department of Mechanical Engineering, University of Minnesota, Minneapolis, Minnesota, USA; 2 Department of Mechanical Engineering, University of California, Berkeley, California, USA

## Abstract

There is an urgent need for sensors deployed during focal therapies to inform treatment planning and *in vivo* monitoring in thin tissues. Specifically, the measurement of thermal properties, cooling surface contact, tissue thickness, blood flow and phase change with mm to sub mm accuracy are needed. As a proof of principle, we demonstrate that a micro-thermal sensor based on the supported “*3ω*” technique can achieve this *in vitro* under idealized conditions in 0.5 to 2 mm thick tissues relevant to cryoablation of the pulmonary vein (PV). To begin with “*3ω*” sensors were microfabricated onto flat glass as an idealization of a focal probe surface. The sensor was then used to make new measurements of ‘k’ (W/m.K) of porcine PV, esophagus, and phrenic nerve, all needed for PV cryoabalation treatment planning. Further, by modifying the sensor use from traditional to dynamic mode new measurements related to tissue vs. fluid (i.e. water) contact, fluid flow conditions, tissue thickness, and phase change were made. In summary, the *in vitro* idealized system data presented is promising and warrants future work to integrate and test supported “*3ω*” sensors on *in vivo* deployed focal therapy probe surfaces (i.e. balloons or catheters).

Focal energy based therapies have a long history of use in the treatment of cancer, cardiovascular and neural disease^1^,^2^,^3^. As the technique evolves, there are increasingly thin and complex tissue anatomies where focal therapies and freezing are being applied that require sub mm monitoring accuracy to avoid debilitating side effects. For instance, cryogenic approaches to treatment of atrial fibrillation are increasingly being used in the pulmonary vein (PV) which is only 1–2 mm thick^4^,^5^,^6^,^7^. This treatment in thin PV often suffers from over and under-freezing suggesting a need for better monitoring. Unfortunately, traditional clinical imaging has difficulty monitoring at or below the mm scale, which is of the order of thickness of the PV itself. For this reason it is not surprising that in a pilot computed tomography (CT) study, we were unable to visualize the PV or the freezing process within it^8^. So, despite the past successes of clinical image guidance for cryoablation in cm sized tissues^9^,^10^,^11^, clinical imaging alone lacks the spatial resolution to successfully monitor freezing in the PV or other thin tissue structures (1–2 mm thick).

Thus, there is an urgent need to monitor tissue contact, thickness and freeze completion during treatment which relatively low-resolution clinical imaging cannot provide at the millimeter to sub millimeter scale. Here we provide evidence that a future solution could involve a micro- thermal sensor based on the “3ω” technique of thermal conductivity measurement (Fig. 1)^12^. The sensor comprises a thin metallic heater line, typically made of gold, and deposited on a flat glass substrate (for additional experimental details, see the Methods section below). By application of alternating electric current to the heater line, thermal waves can be produced which penetrate to a depth (PD) that is inversely proportional to the square root of the electrical current frequency, ω^(1/2)^. From the output signal of the sensor, the average thermal conductivity ‘k’ of the volume traversed by the thermal wave could be obtained. This technique has been commonly used to measure thermal conductivity of inorganic materials^13^,^14^,^15^,^16^. Recently, it has been modified to measure ‘k’ of biological tissues^17^ and even cells^18^. The current article presents proof of principle data to show the ability of the sensor to give thermal conductivity for treatment planning and then extends the sensor use for other measurements relevant for monitoring. For instance, we use the sensor to make the first measurements of ‘k’ of pulmonary vein and the surrounding phrenic nerve and esophageal tissue to prove its ability for use with these thin (<3 mm) cardiac tissues. Next, we modify the sensor use to demonstrate contact with tissue vs fluid (i.e. water), fluid flow conditions, tissue thickness ‘τ’, and freeze completion in fluid or tissue systems. This was achieved by integrating the sensor onto an idealized probe surface (i.e. a flat glass substrate) and testing through *in vitro* experiments. In future, with proper characterization and calibration, the sensor could potentially be integrated onto flexible and stretchable probe surfaces for *in vivo* use (Eg: Cryoballoons and cryoprobes)^19^,^20^,^21^,^22^.

## Results

### Thermal conductivity measurement of thin cardiac tissue

Figure 2 shows the first *k* measurement of porcine pulmonary vein, phrenic nerve, and esophagus by 3ω sensors with typical dimensions of heater line length, L = 1.5 to 2.5 mm and width, b = 30 to 80 μm. The protocol for sample handling is explained in Methods section. During phase change the *k* increases by roughly a factor of 2 in all tissues upon freezing due to ice formation. This will be important for measurement of the onset and completion of freezing as will be explained later.

### Sensing Contact

Figure 3(a–c) shows the schematic of the experimental water flow vs. tissue contact setup and the corresponding change in the 3ω out of phase output voltage, V_3ω,op_ from the sensor (L = 2.5 mm, b = 50 μm). A constant V_3ω,op_ is obtained for stagnant water (0 to 42 s). As water flows (from 42 s), V_3ω,op_ drops due to convective heat loss at the heater line. After placing a piece of pulmonary vein (1 mm thick) on top of the sensor at 64 s, it blocks the water flow and thereby increases the V_3ω,op_ to the highest value of all cases. These results demonstrate that we can sense tissue contact vs. flow. Furthermore, the sensor can measure increases in flow rate directly from V_3ω,op_ as shown in Fig. 3(e). An added benefit is that the signal change can be measured as a function of azimuthal angle (Φ) of the flow, where the signal is maximum for Φ = 90 °C to the sensor. These effects can be explained by laminar convective flow over a flat plate as described in Supplementary Information (SI 4).

### Sensing Tissue Thickness

Two experiments were conducted using a 3ω sensor (L = 2 mm, b = 30μm). For the case of varying backside boundary condition and fixed sample thickness (Fig. 4(a)), at low frequencies (zone (i)), V_3ω,op_ increases for insulated condition and vice versa for fixed temperature condition. In the next experiment (Fig. 4(b)), the backside boundary condition was air at −55 °C, closely approximating an adiabatic boundary. Figure 4(b) shows V_3ω,op_ to be independent of thickness in the zone (ii) where the frequency is high enough for the thermal penetration depth (PD) to be within the sample. Below a characteristic frequency (zone (i)), the measurements become very sensitive to the backside boundary condition, leading to an abrupt increase in V_3ω,op_ as a function of the thickness. This thickness dependence can also be appreciated by plotting V_3ω,op_ at a single low frequency such as 0.1 Hz as shown in Fig. 4(b) (inset). The PD of thermal waves can be calculated by PD = (D/2.ω)^0.5^, where D is thermal diffusivity (m^2^/s)). At 0.1 Hz, the PD into the frozen agargel is ~1 mm. A comparison of an idealized theoretical calculation^17^,^23^ with the experiment shows that the experimental measurements are consistent with the model trends, and suggest that with proper calibration can be used to measure thickness.

### Sensing Phase Change

Two freezing trials were conducted, one with water (Fig. 5a), and one with mouse liver (Fig. 5b) using a 3ω sensor (L = 1.5 mm, b = 80 μm). Both trials show the same overall pattern, with a region of stable V_3ω,op_ at the beginning of the trial, corresponding to when the water droplet is completely liquid. The temperature of the stage below the sensor was changed to −10 °C at ~120 s (~150 s for mouse liver), after which there is a drop in V_3ω,op_. This is followed by a region of stable voltage as the droplet is completely frozen from 120 s to 340 s (150 s to 550 s for mouse liver). This stable region has a lower V_3ω,op_ value than the region corresponding to unfrozen water (Fig. 5a) or tissue (Fig. 5b). This is due to the higher *k* of ice/frozen tissue than water/thawed tissue (Fig. 2). After 340 s (~550 s for mouse liver), the temperature stage is changed to a set point of 20 °C, followed by a region of changing voltage. The graph ends with a third stable region, corresponding again to unfrozen water/tissue. The voltages at the beginning and the end of the above data match closely (to within 3%), showing that water V_3ω,op_ measurements are repeatable. In both cases, the stabilized voltages between the unfrozen water and solid ice differ by at least 35%. The measurement noise, taken as the standard deviation of V_3ω,op_ of frozen liver between 400 and 500 s in Fig. 5(b), is ~0.1 μV which is an order of magnitude smaller than the signal change due to freezing/thawing (i.e., ~5 μV) . Furthermore, a theoretical prediction of the behavior of V_3ω,op_ during a forced change in ‘k’ of a water droplet at 0 °C shows a similar trend showing the ability to sense phase change (Fig. 5(c)). The thickness of ice (τ_ice_), which is characterized by normalized ice thickness (τice/τtotal), grows from the surface below and the ice–water interface is represented by an isotherm of 0 °C. It is important to note that the theoretical prediction of change in V_3ω,op_ is based only on change in ‘*k*’ and the model does not account for the effect of exothermic heat release during freezing.

## Discussion

**Figure 1:**
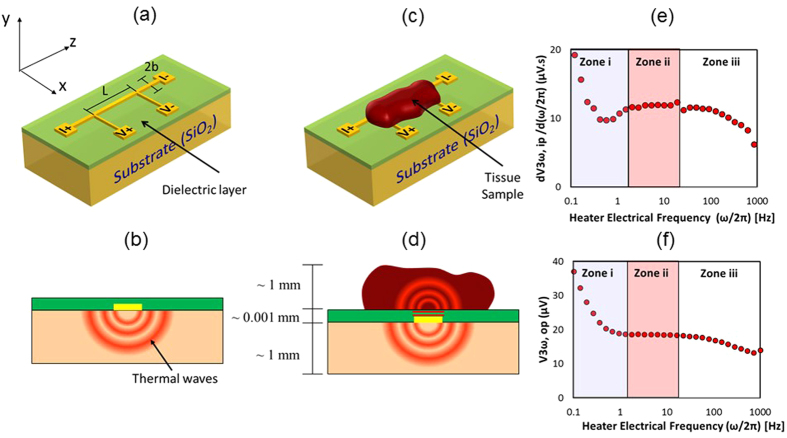
Concept of the supported 3ω method to measure thermal conductivity, ‘*k*’ of thin (>0.1 mm) tissues^17^. (**a**) Supported 3ω sensor comprises a metal heater line with typical dimensions of b = 30 to 80 μm wide, L = 1.5 to 2.5 mm long, ~200 nm thick, microfabricated onto a glass substrate and coated with a dielectric layer. (**b**) Side view (xy plane) of the sensor showing calibration to determine k_sub_. (**c**) Tissue is placed on top of the sensor to measure its k. (**d**) Side view (xy plane) showing thermal waves penetrating into the tissue and the substrate. (**e,f**) Data from measuring *k* of ~1 mm thick frozen agargel at −25 °C with air as a backside boundary condition of the sample. (**e**) Third harmonic voltage slope (dV_3ω,ip_/d(ω/2π)) and (**f**) V_3ω,op_ as a function of frequency of driving current ‘(ω/2π)’; ip is the “in phase” component of the signal, and op is the “out of phase” component. Zones (i) and (iii) refer to the frequency range where the assumptions of Eqns (3) and (4) are not satisfied. Zone (ii) refers to the range where the assumptions of semi-infinite solid and 1D heat source are satisfied.

**Figure 2:**
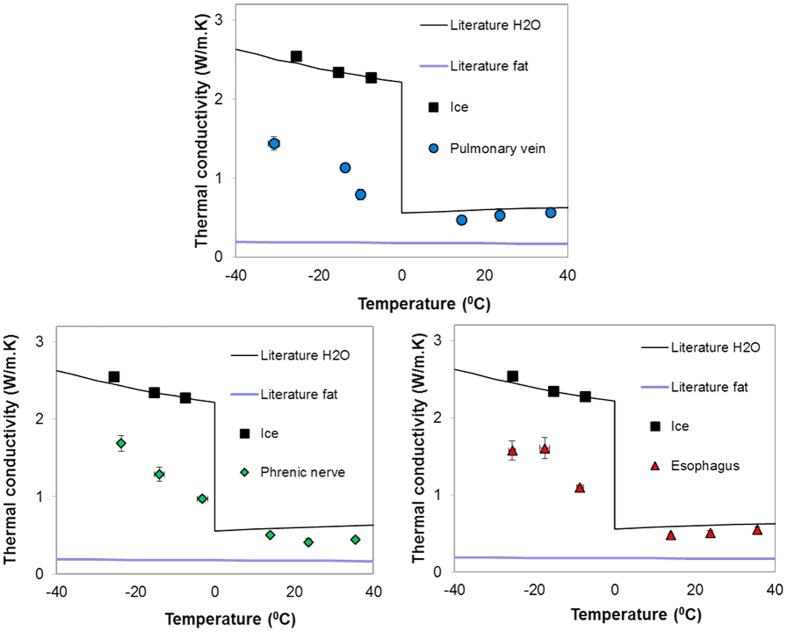
Supported 3ω measurements of porcine PV, phrenic nerve and esophagus. Measurements are the average ± standard deviation of N = 5 samples and validated using ice measurements (black points). The difference in *k* between frozen and unfrozen tissue is roughly a factor of 2. The black and yellow trend-line data are *k* values of water, ice and fat from literature^40^.

In this work we demonstrate the ability of supported 3ω sensors to run in both traditional (steady-state) and a new dynamic mode to measure both thermal properties (traditional use) as well as tissue contact, thickness and phase change relevant to the monitoring of focal therapy in thin tissues. The new “supported 3ω” approach (Fig. 1), which is a variant of traditional 3ω technique^12^,^24^ can measure ‘*k’* within thin tissues (as low as 0.1 mm)^17^ and even cells^17^,^18^. For the first time, this study uses this approach to measure the thermal conductivity of thin cardiac tissues (<2 mm) such as porcine pulmonary vein, esophagus and phrenic nerve for focal therapy treatment planning (Fig. 2). These properties will be necessary to thermally model focal therapies (i.e. treatment planning) within the pulmonary vein and surrounding tissues and are currently not available in the literature^25^. In addition, we show a new use of the sensor based on V_3ω,op_ to detect tissue contact, thickness and phase change. Importantly, V_3ω,ip_ can also be used to sense contact, thickness, and phase change although requiring multiple frequencies and thus longer times which make it unattractive for a focal therapy application (See SI).

**Figure 3:**
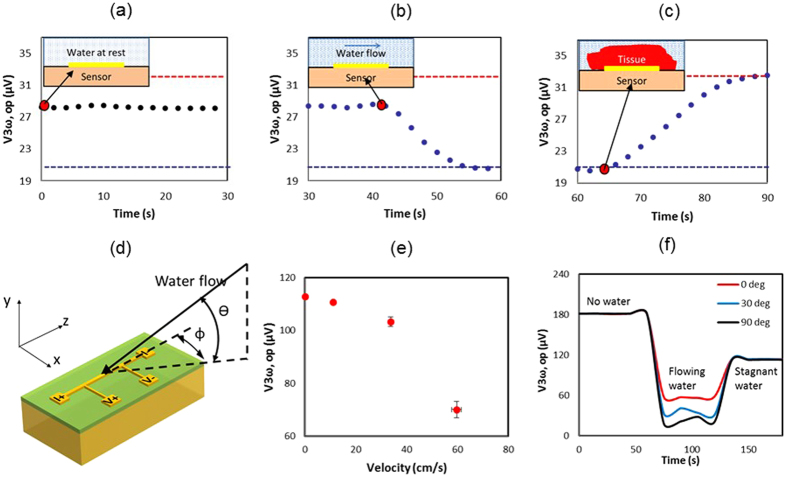
The measurement of flow vs. tissue contact by 3ω sensor. Plots in (**a–c**) show three cases with the schematic inset (x-y plane) for the events: (**a**) water at rest; (**b**) water flowing; and **(c**) tissue in contact with 3ω sensor. Red dots represent the beginning of these events. In all cases the resulting V_3ω,op_ measured from a representative experiment is shown on the y-axis in μV showing that the signal of flowing water <water at rest <bio-tissue using dashed lines of data as visual guides. In the bottom row (**d–f**), graphics in (**d**) show a water flowing past the sensor at an azimuthal angle ‘φ’ and polar angle ‘
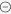
’. Further, (**e**) shows V_3ω,op_ correlates with flow rate for φ = 90 °C, 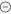 = 0 °C, while (**f**) shows V_3ω,op_ correlates with a variable ‘φ’, under fixed ‘
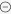
’ of 0 °C and flow rate of 20 cm/s (with some oscillations between 60 s and 120 s due to transient behavior of the pump). Data in (**e,f**) were obtained with a different input current of 30 mA. Data in (**e**) is the average ± standard deviation of N = 5 samples. Horizontal error bars are less than 5%.

**Figure 4:**
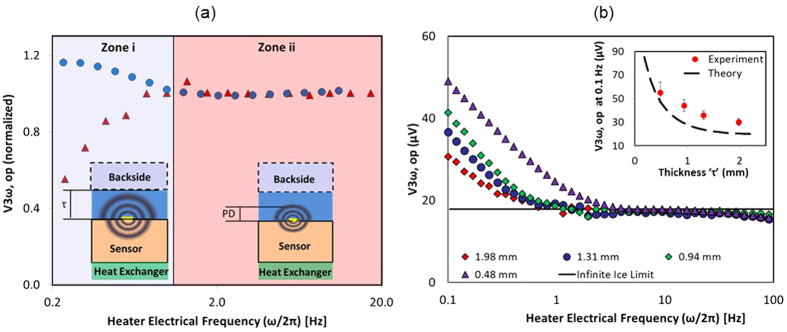
The measurement of thickness by 3ω sensor. (**a**) Plot shows a representative experiment of V_3ω,op_ shown in y-axis, where at low frequencies in zone (i), it depends on backside boundary for fixed sample thickness (τ ≈ 0.5 mm). Blue circle data represents the case where backside of the sample is low k plastic (quasi-adiabatic) and red triangle data represents the case where backside is high k copper at fixed temperature (quasi-isothermal). Inset schematic shows the comparison between PD and 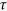 for zones (i) and (ii). (**b**) Plot shows a representative experiment where V_3ω,op_ at low frequencies in zone (i) depends on sample thickness for a fixed backside boundary condition of air. Ice of known thickness (as noted inside figure) is placed on the surface of the sensor at −25 °C. Backside boundary condition is air at −55 °C. Inset shows plot of V_3ω,op_ at 0.1 Hz vs thickness of the sample ‘
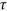
’. Data is the average ± standard deviation of N = 3 samples.

Figure 3 shows the new ability of the sensor to detect contact with tissue vs fluid (i.e. water) with different ‘*k*’. The response time is typically ~15 s as shown in Fig. 3(a–c). Further, by calibrating the reduction in V_3ω,op_ one can also sense different fluid flow rates (as shown in Fig. 3(e)). Once in contact with the tissue, the sensor can be used to measure the thickness of the pulmonary vein (or other thin luminal structure in contact). Thickness measurements exploit the low frequency (PD ≈ sample thickness) regime in zone (i) of Figs 1(e,f) and 4 (a). Thus, the frequency of the electric current can be reduced such that the PD of the thermal wave is comparable to the thickness of the sample. At this point, the signal becomes sensitive to the backside boundary condition as shown in Fig. 4(a) zone (i). Fig. 4(b) inset shows that V_3ω,op_ increases with thickness at lower frequencies, showing an increased sensitivity to the backside boundary condition (in this case air) as PD increases. However, there is a discrepancy in magnitude between the theory^17^,^23^ and experiment despite a similar trend. This difference could be attributed to the complex construction of the sensor (i.e. multi-layered geometry) as compared to the theoretical model. In the experiments, the sensor has a second glass substrate (~1 mm thick) as a physical support, attached with polyimide adhesive (~100 μm thick). Furthermore, the sensor is placed on top of a temperature control stage (~2 mm from the heater line). In addition, there are copper wires and epoxy bonds connecting the heater line to the electronics. This complicates the thermal model, especially at lower frequencies where the relatively long time constants allow deeper PD from this multi-layered sensor geometry. In contrast, theory assumes a simpler, more idealized sensor geometry as depicted in Figs 1(a–d) and 4(a- inset). Despite these differences, a consistent trend between the theory and the experiment provides a qualitative proof that the sensor can measure thickness, and absolute agreement can be explored further in the future.

**Figure 5:**
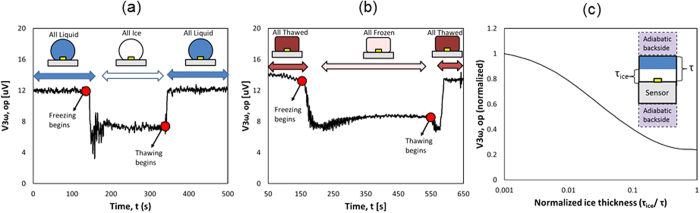
The measurement of phase change onset, completion and thaw by the 3ω sensor. Representative experiments of freezing (**a**) water drop and (**b**) mouse liver freezing is from bottom to top. V_3ω,op_ was measured as a function of time at constant PD (i.e. frequency-1.03 Hz). In (**a**) water was cooled from below at ~120 s and thawed at ~340 s. In (**b**) mouse liver was cooled from below at ~150 s and thawed at ~550 s. (**c**) Theoretical prediction of behavior of V_3ω,op_ at 1 Hz, as a function of normalized thickness of ice as freeze front propagates from below during cooling. Inset schematics are not drawn to scale.

Next, the sensor can be used to sense the phase change in the tissue (Fig. 5). During freezing, the change in ‘k_sample_’ between frozen and thawed tissue (Fig. 2) leads to a change in the V_3ω,op_, and an inverse behavior during thawing. In addition, theoretical predictions in Fig. 5(c) show that V_3ω,op_ is a function of ice thickness as the ice front propagates away from the sensor.

An important aspect of sensing is the need to minimize measurement time, which increases as sensor frequency decreases (i.e., PD increases). Thus, the sensor would require long electronic time constants, especially for several mm thickness measurements to obtain a stable signal. The longest electronic time constant used in this article is 10 s at ~0.1 Hz. In comparison, cryotherapy of PV typically lasts ~120 s^4^ and clinical imaging techniques have temporal resolution of <1 s^7^,^26^,^27^,^28^,^29^,^30^,^31^,^32^,^33^,^34^. Further reduction in the time of measurement and possibly multi-plexing at a variety of frequencies remain interesting and important future improvements for this measurement.

## Methods

### ‘Supported’ 3ω technique

Figure 1 shows the basic concept of the supported 3ω method^17^. A periodic electrical current (~40 mA rms) of angular frequency ‘ω’ is passed through the heater, I = I_0_sin ωt, between the leads I + and I-. This causes Joule heating with a 2nd harmonic component, Q_AC_ = Q_0_sin 2ωt. Thus the heater strip acts like a periodic line heat source producing thermal waves as shown in Fig. 1(b,d). The exact analytical solution to this conduction problem is well known^12^,^17^,^35^. Thermal waves propagate and decay radially away from the heater and PD can be calculated using Eqn (1)


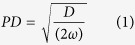


where D - thermal diffusivity (m^2^/s), 2ω- frequency of Joule heating (rad/s). The temperature rise of the heater strip due to Joule heating is inversely proportional to the effective ‘k’ of the system, oscillates at 2ω, and is typically kept smaller than 1 K rms. Subsequently, the temperature oscillation causes the heater’s electrical resistance ‘R’ to also fluctuate at 2ω. Finally, when multiplied by the 1ω current, this 2ω resistance fluctuation causes a component of voltage across the heater to oscillate at the 3rd harmonic. Thus the resulting 3ω voltage (~10 s to 100 s of μV), measured between the leads V + and V- using a lock in amplifier, results in k_total_, which contains information about the known ‘k’ of the substrate, ‘k_sub_’, and the unknown ‘k’ of the sample, ‘k_sample_’. A lock in amplifier is needed to extract 3ω signals of low magnitude from the noisy environment. To determine k_sample_ of a biological tissue, the sample is placed on top of the sensor as in Fig. 1(c,d). The effective thermal conductivity, ‘k_total_’, measured from the 3ω voltage, satisfies Eqn (2) (<1% error)^17^ with k_sub_ known from calibration,


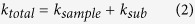


Next is the determination of ‘k_total_’ from the 3ω voltage, which comprises two components - in phase and out of phase. k_total_ can be determined from either of these components as shown in equations (3) and (4).


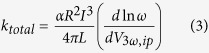



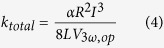


where α - temperature coefficient of resistance (1/°C), L- Length of the heater line (m), V_3ω,ip_ - in-phase component, and V_3ω,op_ - the out of phase component of the 3ω output voltage (V). Eqns (3) and (4) can only be applied in a certain frequency range where their assumptions are satisfied^12^. The first assumption is that the substrate and the sample are semi-infinite, (τ_sub_/PD > 5 and τ_sample_/PD > 5 for 1% error)^23^, which bounds the frequency (∝1/PD^2^) in the lower limit. The next assumption is that the heater is a one dimensional cylindrical heat source (PD/b > 5 for 1% error)^23^ bounding the frequency in the upper limit. For instance, a representative sensor (L = 2 mm, b = 30 μm) was used to measure the ‘k’ of 1 mm thick frozen agargel at −25 °C. In this case, PD should be between 0.08 mm (PD/b > 5) to 0.2 mm (τ_sample_/PD > 5), which corresponds to a frequency range of 2 to 20 Hz (D_ice_/(4π•PD^2^) from Eqn (1)). The raw data in Fig. 1(e) shows that V_3ω,ip_ is linearly proportional to ln (ω/2π) in zone (ii), thus satisfying the assumptions of Eqn (3). The varying slope in the zone (i) indicates that the assumption of semi-infinite solid is violated, while the variation in zone (iii) indicates that the assumption of 1D heat source is violated. Similarly, V_3ω,op_ is independent of frequency in zone (ii), thus satisfying the same assumptions in Eqn (4). Thus the average slope value (dV_3ω,ip_/d(ω/2π)) and the average V_3ω,op_ in zone (ii) is used in Eqns (3) and (4). For traditional 3ω k measurements, the k determined from (dV_3ω,ip_/d(ω/2π)) is the value commonly reported in the literature^12^,^36^ as the slope is independent of contact resistance between the heater line and the sample^13^. Importantly, both V_3ω,op_ and V_3ω,ip_ can be used to sense contact, thickness, and phase change. However, V_3ω,ip_ requires multiple frequencies for thickness measurement and thus longer times making it less attractive for our intended use (See SI).

### Thermal conductivity measurement of thin cardiac tissues

The construction of the sensor is explained in SI 1. Porcine PV, esophagus and phrenic nerve were obtained from sacrificed animals from other IACUC approved studies, immersed in phosphate buffered saline (PBS) and stored in a 4 °C refrigerator^37^. During measurement, the tissue was sliced into approximately 2 mm long samples and placed on the heater line. To avoid dehydration and/or evaporation of water content in the tissue, the sample was encapsulated on the sides and at the top by agar gel (0.5% by weight agarose powder dissolved in water at 65 °C and solidified overnight in a 4 °C refrigerator). The sample- agar gel complex is then covered by a plastic wrap to isolate the sample from evaporative cooling and sublimation which interfere with a consistent and correct measurement^17^. Importantly, in this system, water evaporation or ice sublimation from the tissue was negligible (weight change < 5%) during the measurement in Fig. 2. Finally, all measurements were made within 24 hours after tissue host sacrifice to avoid significant changes in water content^38^.

### Sensing Contact versus Flow

Our experimental setup consists of a supported 3ω sensor (L = 2.5 mm, b = 50 μm) and a variable speed peristaltic pump (Cole-Parmer Masterflex^®^). A velocity range of 10 to 60 cm/s was chosen to mimic blood velocity in cardiac vessels. For sensing tissue contact, PV was manually placed on top of the sensor, thereby blocking the water flow. The sensor recorded the V_3ω,op_ (∝1/k) at fixed frequency of 10.1 Hz (constant PD) and input RMS current of 30 mA. A lock-in time constant of 1 s was selected as a compromise between measurement noise and time response of the measurement system. The chosen frequency is a typical value used to measure ‘k’ of biological tissues (>0.1 mm thick) with temperature above 0 °C (see zone (ii) of Fig. 1(e,f)).

### Sensing Tissue Thickness

Agargel (0.5% weight of agarose in water) was placed on the sensor (L = 2 mm, b = 30 μm) and immediately frozen to −25 °C in a cryofreezer (Planer Kryo 10 Series III). Next, for an input RMS current of 20 mA, V_3ω,op_ was recorded as a function of frequency at a fixed temperature of −25 °C on the sensor surface. First, the backside boundary condition was changed to be thermally insulated (plastic, k ~ 0.2 W/m.K) vs isothermal (Copper, k ~ 385 W/m.K) at −25 °C for constant sample thickness (~0.5 mm). Next, the thickness of frozen agargel was changed from ~0.48 mm to ~1.98 mm while keeping the backside boundary condition as air (k_air_ ~ 0.02 W/m.K) at −55 °C^ ^39.

### Sensing Phase Change

Deionized water was pipetted onto the sensor (L = 1.5 mm, b = 80 μm). The dielectric layer caused the water to bead instead of spread, with the water droplet having an approximate spherical diameter of 2 mm. The temperature was lowered from 20 °C to −10 °C, allowing the water droplet to completely freeze into ice. After allowing the freezing event to complete, and the voltage to stabilize, the temperature was increased back to 20 °C. Completion of freezing and thawing events were verified by visual inspection during the experiment. A measurement frequency of 1.03 Hz was used, having PD in deionized water of 0.46 mm with a lock-in time constant of 1 s. This experimental procedure was repeated on a mouse liver sample (thickness~3 mm) to confirm the behavior in biological tissues.

## Additional Information

**How to cite this article**: Natesan, H. *et al.* A Micro-Thermal Sensor for Focal Therapy Applications. *Sci. Rep.* **6**, 21395; doi: 10.1038/srep21395 (2016).

## Supplementary Material

Supplementary Information
