# Supplementary material for: A Micro-Thermal Sensor for Focal Therapy Applications
Source: Sci Rep. 2016 Feb 26;6:21395. doi: 10.1038/srep21395 (PMC4768245; doi:10.1038/srep21395)
Supplement: Supplementary Information [file srep21395-s1.pdf]

A Micro-thermal Sensor for Focal Therapy Applications

Harishankar Natesan,[a] Wyatt Hodges,[b] Jeunghwan Choi,[a,c] Sean Lubner,[b] Chris Dames[b] and John Bischof*[a]

[a] Department of Mechanical Engineering, University of Minnesota, Minneapolis, Minnesota, USA

[b] Department of Mechanical Engineering, University of California, Berkeley, California, USA

[c] Department of Engineering, East Carolina University, Greenville, North Carolina, United States

† Jeunghwan Choi's works related to this article were carried out in University of Minnesota. East Carolina University is his current address.

Supplementary information 1:

Construction of the supported 3ω sensor:

The advantage of supported 3ω sensors is summarized in Table S1. Using standard

photolithography and liftoff microfabrication techniques, a gold heater line, with typical dimensions of 30 μm wide, 1 to 3 mm long, and 200 nm thick, is deposited on a glass substrate (1 mm thick amorphous SiO_2). A 5 nm thick Chromium adhesion layer holds the gold heater line and glass together. The heater lines are connected to the signal processing unit and a lock in amplifier (SR 830, SRS, Sunnyvale CA) using copper wires. As the soft, hydrated biological tissue is weakly electrically conducting, a thin dielectric film is incorporated between the heater and the sample to prevent electrical cross-talk, after connecting the wires to the heater line. This was achieved by dissolving polystyrene pellets in toluene at a concentration of 15 mg/mL. A few drops of the solution were dropped directly on top of the sensor, which was then held vertical to allow excess solution to run off. The toluene evaporates in 5 to 10 seconds leaving a thin continuous layer (~ 1μm) of polystyrene dielectric layer (Fig 1(a, b)).

Supplementary information 2:

(a) Theory behind flow rate measurement:

The theory behind flow rate measurement is that the electrical power input to the heater is lost as heat by conduction to the substrate below and convection to the flowing water above the sensor as given by Eqn (S1).

$$Q = I^2 R = h_{conv} A\left(T_{hl} - T_f\right) + G_{cond}\left(T_{hl} - T_\infty\right) \qquad \text{(S1)}$$

where Q - Joule heating power input to the heater line (W), h_{conv} - convective heat transfer coefficient (W/m^2.K), G_{cond} - thermal conductance of the substrate (W/K), A - footprint area (L x b) of the heater line (m^2), T_{hl} - temperature of the heater line (K), T_f - temperature of the fluid (K),

and T_∞ - ambient environment temperature (K). The variables in Eqn (S1) can in general be complex and time-dependent. If current 'I' and the substrate thermal conductance 'G_{cond}' are

Measurement Characteristics	Current techniques*	Traditional 3ω	Supported 3ω
Can measure sizes < 1 mm	×	✓	✓
Compensates for Parasitic Heat Loss	×	✓	✓
Compensates for Contact Resistance	×	✓ (only for in – phase)	✓ (only for in – phase)
Use in Biomaterials	✓	×	✓
Reuse possible	✓	×	✓
Avoids Microfabrication	✓	× (necessary for every sample)	× (necessary for one sensor only)

Table S1. Comparison of Thermal Conductivity Measurement Techniques. *The 'Current Techniques' refers to a representative sample of techniques (Guarded Hot plate, Embedded Thermistor, Cut bar) (Reproduced from Lubner et al *IMECE* 2012 with Copyright (2012) Permission from ASME)[1]

maintained constant, then changes in convective heat loss (h_{conv}) arising from changing the flow will result in changes in the temperature at the heater (T_{hl}). This will be reflected as changes in 'R', and thus $V_{3\omega, op}$. Thus the sensor can be used to sense flow rate in a similar fashion to other past approaches such as hot wire anemometry.[2,3]

(b) Dependence of 3ω signal on flow direction:

Figure 3(f) shows the signal change depends on the azimuthal angle (ϕ) of the flow direction. These effects could be explained by laminar convective flow over a flat plate with an unheated starting length, as shown in the Eqn (S2)[4]

$$h_{conv} = \frac{\overline{Nu}_x k_{water}}{x} = \overline{Nu}_x\Big|_{\xi=0} \frac{k_{water}}{x-\zeta}\left[1-\left(\frac{\zeta}{x}\right)^{\frac{3}{4}}\right]^{\frac{2}{3}} \qquad \text{(S2)}$$

where 'x' is the total distance travelled by water on the substrate. 'ζ' is the unheated starting length, referring to the distance travelled by water over unheated glass substrate and 'x-ζ' can be referred as the heated length. In the case of the sensor, heated length is a function of flow angle 'φ'. For $\varphi=0^0$, the heated length will be 'L', i.e., the thermal boundary layer will develop over the length of the heater line 'L'. In contrast, for $\varphi=90^0$, the heated length 'x' is the width of the heater line 'b' plus the thermal penetration depth (PD). It is known from Eqn (S2) and the boundary layer theory that the average convection coefficient (h_{conv}) will decrease with increase in boundary layer thickness[4]. As (b+PD)<<L, thermal boundary layer thickness will be small across the heater line. Thus h_{conv} will be higher for the case $\varphi=90^0$, thus explaining the behavior in Figure 3(f).

Supplementary information 3:

Use of in phase 3ω signal ($V_{3\omega, ip}$) to sense contact, thickness and phase change:

Figure S1 shows the use of $V_{3\omega,\,ip}$ to sense contact, thickness and phase change. The sensor is

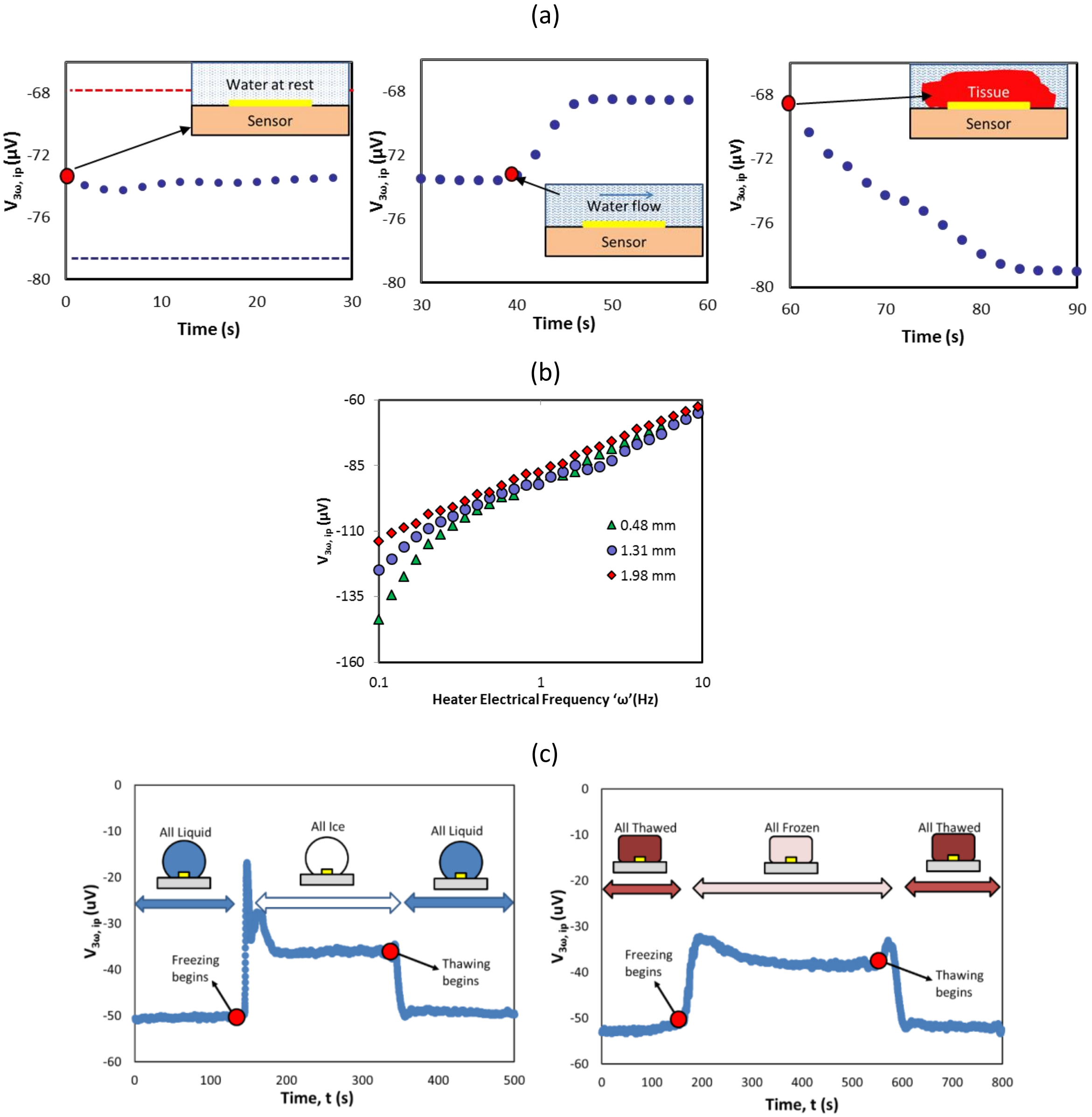

Figure S1. Use of in phase 3ω signal ($V_{3\omega,\,ip}$) to sense (a) contact, (b) thickness and (c) phase change *(a) Sensing contact:* Plots in (a) show three cases with the schematic inset (x-y plane) for the events, water at rest, water flowing, and tissue in contact with 3ω sensor. Red dots represent the beginning of these events. *(b) Sensing thickness:* Plot shows that $V_{3\omega,\,op}$ at low frequencies (<~0.5 Hz) depends on sample thickness for a fixed backside boundary condition of air. Ice of known thickness is placed on the surface of the sensor at -25^{0}C. Backside boundary condition is air at -55^{0}C. *(c) Sensing Phase change:* Representative experiments of freezing water drop and mouse liver freezing is from bottom to top. $V_{3\omega,\,op}$ was measured as a function of time at constant PD (i.e. frequency-1.03 Hz). Water was cooled from below at ~120 s and thawed at ~340 s. Mouse liver was cooled from below at ~ 150 s and thawed at ~550 s;

run in “dynamic” mode (i.e. at a single frequency as a function of time) to sense contact (Figure S1(a) and phase change Figure S1(c). The sensitivity and response time is similar to that of out-phase signal discussed in the main paper. However for thickness measurement (Fig S1(b)), the backside boundary is sensed at a lower frequency (~0.5 Hz) for in phase signal. In comparison, the backside boundary is sensed at a higher frequency (~0.7 Hz) for out of phase signal. Thus out of phase signal has the potential for faster measurement.

References

1 Lubner, S. D. *et al.* in *ASME 2012 International Mechanical Engineering Congress and Exposition.* 1397-1404 (American Society of Mechanical Engineers).

2 Lomas, C. G. *Fundamentals of Hot Wire Anemometry*. (Cambridge University Press, 2011).

3 Bruun, H. H. Hot-Wire Anemometry: Principles and Signal Analysis. *Meas Sci Technol* **7**, null, doi:10.1088/0957-0233/7/10/024 (1996).

4 Incropera, F. P. *Fundamentals of Heat and Mass Transfer*. (John Wiley & Sons, 2011).
